# Reframing Professionalism: The Virtuous Professional

**DOI:** 10.15694/mep.2018.0000074.1

**Published:** 2018-04-03

**Authors:** William Wadland, Margaret Thompson, Donna Mulder, Tom Tomlinson, Steven Roskos, John Foglio, John Molidor, Janet Osuch

**Affiliations:** 1 Michigan State University

**Keywords:** Professionalism, Faculty Development, Medical Student

## Abstract

This article was migrated. The article was marked as recommended.

In response to prevalent unprofessional behaviors during the 1990s, the medical school administration at Michigan State University’s College of Human Medicine developed a student curriculum for professional development, called “The Virtuous Student Physician.” However, as students adopted these professional aspirations and attributes, they noted that faculty members were not being held to the same standards.

The medical school’s senior associate dean for faculty affairs and development convened a task force to reframe professionalism for all faculty, residents, and students. Our first step was to survey our faculty regarding their awareness of the student professionalism curriculum and their own perceived professional weaknesses. This survey showed the following: most faculty members were aware of “The Virtuous Student Physician” curriculum, that faculty members identified social responsibility as the most difficult attribute to achieve, and that the most difficult behavior identified was working to resolve problem behaviors with colleagues.

The task force then developed a new curriculum “The Virtuous Professional: A System of Professional Development for Students, Residents, and Faculty.” The task force identified three core virtues (Courage, Humility, and Mercy) and reframed the professional attributes encompassed by these virtues to be aspirational for the entire learning community. The faculty of the College subsequently adopted the new principles and practices, including the use of routine, anonymous student evaluation of faculty professionalism.

We are currently collecting data from student evaluations of their clinical faculty members. We plan to use this feedback to guide faculty development and recognize those who model exemplary professionalism as well as to address those who engage in unprofessional behavior.

## Introduction

The College of Human Medicine (CHM) at Michigan State University (MSU) was founded in 1965 with a mission to “serve the people” of the State of Michigan by training future physicians in the context of state-wide communities and with “the understanding of man as a biological and psychological unit, sharply responsive to ecological and sociological forces [ Hunt 1990].” To fulfill this vision, the founding dean of CHM, Andrew Hunt, recruited faculty in both medical education and humanities to complement the faculties at MSU in the basic sciences [ Hunt 1990]. He developed an Office of Medical Education Research and Development (OMERAD) and a Center for Medical Ethics and Humanities in the Life Sciences [ Andre, Brody et al. 2003]. The inaugural curriculum of the College integrated ethics and humanities with basic science and clinical skills with a focus on “problem-based learning” and on the patient with “focal problems [ Hunt 1990].” Post-graduate surveys of residency program directors have routinely given CHM graduates high marks in communication skills, successful community membership, and professional attributes [ OMERAD 2015].

In 1991, in response to blatant breaches in professional behavior by students, such as open sharing of test questions and excessive tardiness during clinical clerkships, the College convened a task force to address these behaviors. The result was a comprehensive student curriculum: “The Virtuous Student Physician.” This required curriculum involved all four years of medical school (refer to Table 1 for specific courses and activities). Six attributes framed the curriculum: 1) competence, 2) honesty, 3) respect for others, 4) compassion, 5) professional responsibility, and 6) social responsibility. Assessment of professional behavior of students occurred in the pre-clinical program and clinical clerkships with multiple opportunities for “reflecting, talking, and acting” [ Hoppe 2001]. Demonstration of professionalism was a written graduation requirement.

Over time, numerous complaints by students arose concerning faculty (and resident) lapses in professionalism, such as severe tardiness or verbal abuses of students and colleagues. Students complained of being held to higher standards than the faculty or residents instructors. Without holding our faculty to the same professional expectations as our students, we seemed to be fostering “the hidden curriculum” of adverse faculty mentoring of unprofessional behavior [ Hafferty and Franks 1994; Hafler, Ownby et al. 2011]. In 2008, the College convened a new task force to reframe professionalism to be all inclusive of students, residents, and faculty.

This report of “Reframing Professionalism: The Virtuous Professional” describes the work of the task force focusing on: 1) the assessment of the knowledge, participation, and expectations of the MSU-CHM faculty concerning medical student professional development; 2) the process of reframing the core values of professionalism to be highly apparent for the entire learning community; and 3) the processes to foster faculty engagement, development, and accountability on professionalism.

## Assessing Faculty

### Survey Overview and Methods

In fall of 2009, the task force developed and surveyed faculty, either paid directly by MSU-CHM or indirectly by one of our affiliated community programs, to assess their knowledge and familiarity with the professionalism curriculum and the six attributes taught throughout all four years of the medical student program. The survey was approved as ‘exempt’ status by the Institutional Review Board of Michigan State University.

The survey queried faculty about their involvement in the professionalism curriculum, how challenging the six attributes were to achieve or portray as a faculty member, and their interest in faculty development regarding professionalism.

There was a total of fifty-two questions on the survey. Twenty-nine of the questions related to the indicators of professional development under each of the six attributes. Each item was scored using a 4 point Likert scale. A summary rating was generated for each indicator with a simple average and standard deviation.

We asked faculty to rate the six attributes and associated behaviors that they felt were most challenging to actualize as an educator. We also asked faculty to rank strategies for educating faculty about the professionalism attributes and curriculum.

### Results of Faculty Self-Assessment of Professional Virtues and Behaviors

Of the 548 faculty who received the survey, 285 responded (52 percent). Of those faculty who completed the survey, 62 percent reported that they were familiar with the professionalism curriculum. A majority, however, reported that there were limited opportunities to participate as faculty in related courses and activities ( Table 1).

**Table 1.** Faculty Engagement in Student Professionalism Curriculum

Student Professionalism CurriculumCourses/Activities	Faculty FamiliarityN=203	Faculty Participation (Presenter/ Educator)N=177
Pre-Matriculation Workshop	29%	8%
Core Competences on Virtuous Physician	46%	10%
Professionalism Student Log	33%	13%
Social Context of Clinical Decisions/ Ethics Course	45%	17%
Introduction to Physician-Patient Relationship Course	55%	24%
Longitudinal Patient-Centered Experience	51%	28%
White Coat Ceremonies	74%	31%
Clinical Performance Student Evaluation	51%	31%
Clinical Skills Expectations	65%	35%
Required Course Evaluations	55%	36%
Mentor Program	75%	41%
Small Group Evaluations	59%	59%

Faculty rated the following attributes from the most challenging to the least challenging: Social Responsibility (2.31, SD 0.90), Professional Responsibility (1.99, SD 0.86), Compassion (1.83, SD 0.78), Competence (1.82, SD 0.82), Honesty (1.58, SD 0.76), and Respect for Others (1.58, SD 0.69). Faculty rated the following professional behaviors from most challenging to least challenging: Working to resolve problem behaviors involving colleagues (2.47, SD 0.84), Addressing social factors that adversely affect the health of patients (2.31, SD 0.90), Seeking feedback on the effects of my behavior on others (2.30, SD 0.76), Addressing personal limitations-other barriers to learning and growth (2.19, SD 0.80), Demonstrating conflict resolution in a collegial manner (2.03, SD 0.79), and Accurately reporting actions and events of dishonesty (1.83, SD 0.89).

Faculty ranked the following strategies for faculty development from most to least useful: New faculty orientation (2.85), Feedback in annual review process (2.62), Faculty retreats (2.47), Grand rounds (2.43), Web instruction (2.29), Appreciative listening groups (2.23), Faculty handbook (2.09), Role-play exercises at faculty meetings (1.96), and Posters (1.53).

## Reframing and Developing the CHM Core Virtues

In the first meetings of the task force, it quickly became apparent that student concerns about the double standard being applied to faculty was not the only issue deserving attention. There was something fundamentally lacking in the original professional “virtues” or attributes (i.e., competence, honesty, professional responsibility, respect for others, compassion, and social responsibility) themselves. By and large, they were not virtues but rather they were basic responsibilities.

To start with an obvious one, “competence” is rightly expected of every student and every physician. It defines a baseline or floor that sits somewhere well below “excellence.” Those who are not competent should not graduate or practice. Or take “honesty.” There are minimal expectations of honesty to which all students and physicians are rightly held. One of the “indicators” for possessing the virtue of honesty was that “A student striving for honesty will accurately report actions and events and avoid cheating, plagiarism, and misrepresentation of the truth.” A student who cheats is not merely failing to “strive” for honesty-he’s being dishonest.

Nearly all the original “virtues” were operationalized in similar ways (although compassion was the lucky exception). With the demands of professional virtue reduced to avoiding such obvious transgressions, “professionalism” seemed to students to be hardly worth talking about-and certainly not at length. When combined with a system that dinged students for a “professionalism lapse” for being five minutes late for class, “professionalism” became a dirty word.

What had been lost was the idea that virtues require more than merely meeting our responsibilities. It is, of course, vital to avoid unethical behavior toward patients, colleagues, and others, but that is a minimal expectation. In the larger context of the pursuit of excellence, it is also essential to aspire to ideals that reach beyond doing the right thing toward becoming the kind of person and the kind of professional we would most like to be.

Properly understood, virtues are aspirational and difficult to achieve. They are also important in that they give meaning to our actions, even when we are merely doing what we are supposed to do. To act from *mercy*, for example, we need to know when others are suffering. This is not an intellectual knowledge. It arises out of our own feelings, which are triggered by and resonate with the other person’s feelings. Without it, we can act in “compassionate” ways because we know our role requires it, but our compassion will not reflect any virtue. It will only be performance of a duty.

Virtues also support acting on our responsibilities. Being truly respectful of others, for example, requires some *humility.* In this case, humility about the truth and certainty of our own beliefs and values is vitally important. This is necessary for cultivating a tolerant open-mindedness. It is easy to respect those who are like us. It is difficult to respect those who are different. Without the virtue of humility, outwardly respectful behavior becomes grudging toleration.

*Courage* is another supportive virtue. Being honest requires courage when telling or acting on the truth runs some personal risk of retaliation by those who have power.

So for these reasons, we created a framework of three virtues: **C**ourage, **H**umility, and **M**ercy. These, of course, are not the only virtues that might be relevant to being an excellent health professional. However, they are more than adequate to define an aspirational context in which the original responsibilities are understood. They also invite discussions of professionalism, among students and faculty, which move beyond the obvious into professionalism territory that tests us intellectually and emotionally, which is where real professional growth happens. The virtues of **C**ourage, **H**umility, and **M**ercy, were intentionally chosen to match the abbreviated letters of the College of Human Medicine (CHM) in order to promote easy recall and application by faculty and students.

**Figure 1.**
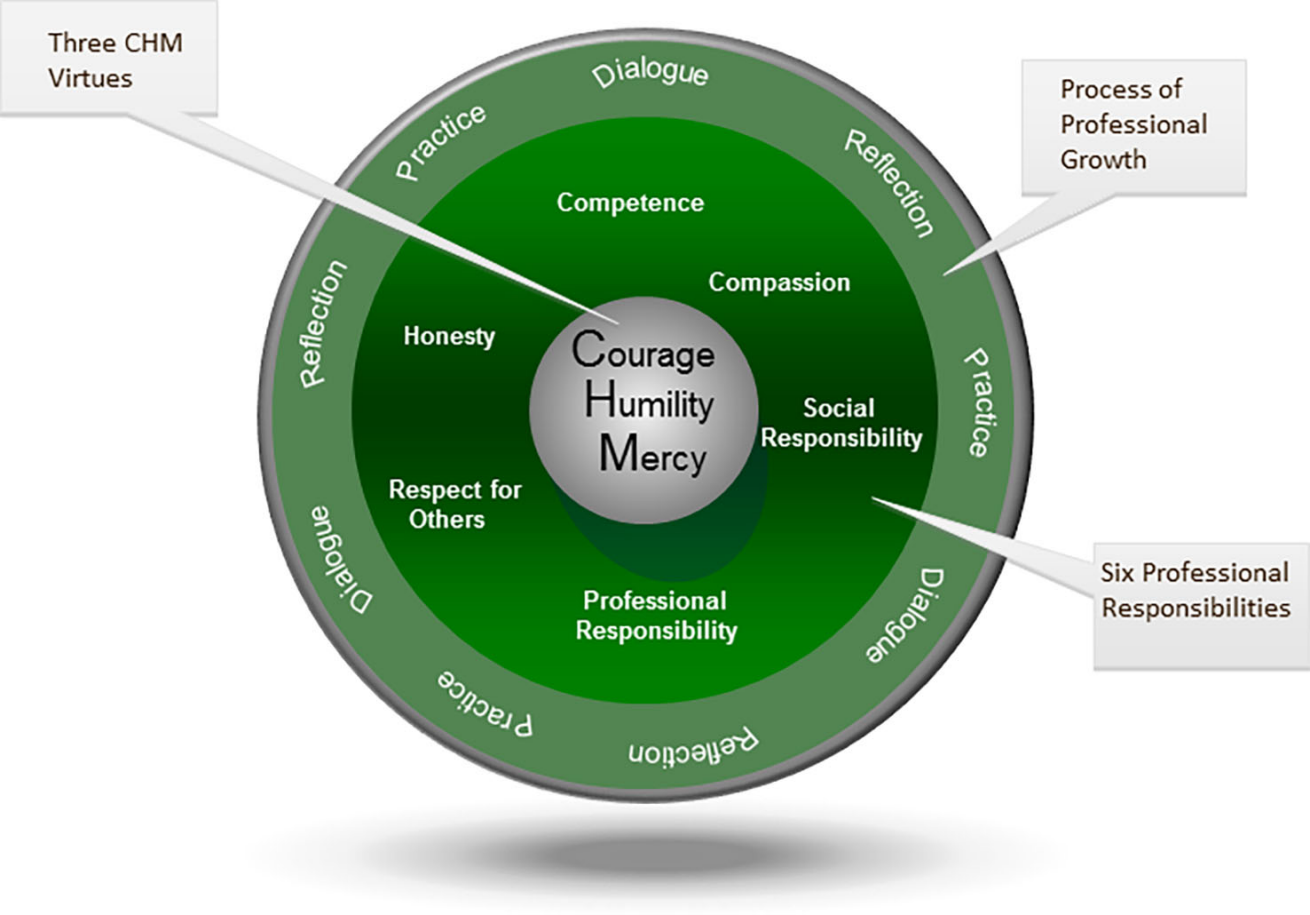
The Virtuous Professional

In order to operationalize the process of professional growth and the process of aspiring to these three virtues, we use the three modes of dialogue, reflection, and practice. Throughout the virtuous professional curriculum, students and faculty are asked to reflect upon and discuss issues related to the virtues and responsibilities in the context of clinical care and to help each other to understand how to practice these attributes ( Figure 1).

## Developing the Professional Standards and Responsibilities

We identified specific minimum standards of conduct, below which a CHM professional should not fall. These are qualities to which every physician should aspire and define conduct to avoid. We operated under three important principles:

1. Our efforts would focus on the educational setting exclusively. Since we are a community-based medical school with hundreds of faculty at dozens of different sites throughout the state, this principle acknowledged that the College has a fiduciary responsibility to our students, but the hospital system to which a given faculty member belongs has its own set of standards and policies in non-education contexts over which the College possesses no oversight.
2. Residents play a critically important role in the teaching environment. We therefore expanded our approach to include three important professional groups: students, residents and fellows, and attending physicians.
3. The expectations of attending faculty will differ from those of residents and students, as professional growth is an iterative process, and role-modeling and mentorship is a recognized responsibility.

We began with a literature review and a review of the professional standard documents of several organizations. We incorporated what we perceived as best practices into a framework based on the six attributes of the original Virtuous Medical Student document and expanded it to apply to the three groups of professionals referenced above when appropriate.

The results of the work of the task force was a final professionalism document “The Virtuous Professional: A System of Professional Development for Students, Residents and Faculty.” http://www.chmfacultyaffairs.msu.edu/professionalism/VP.pdf

## Fostering Faculty Engagement

We tackled the problems of creative dissemination and training of faculty in the newly formulated virtues and professionalism curriculum. We developed a brief presentation for faculty highlighting: 1) the need for comprehensive professionalism standards for students, residents, and faculty, 2) the results of the faculty survey, 3) the process for reframing professionalism for the College, and 4) the need for faculty to be held accountable to professional behaviors similar to students. We also developed several clinical cases to spark discussion for practical application of the new virtues of Courage, Humility, and Mercy to real life situations ( Table 2).

**Table 2.** Cases for Presentation to Illustrate New Model

Case	Attributes Demonstrated	Virtues Demonstrated	Audience
**Student complains about faculty member using off-color, sexist, or racist cartoons in a PowerPoint lecture. Faculty member must address with colleague**	• Honesty • Social Responsibility • Professional Responsibility • Respect for others	• Courage	• Faculty • Department chairs • Students
**Student concerned about hospital staff comments regarding same sex couple with new baby at risk for neonatal withdrawal syndrome**	• Honesty • Compassion • Respect for others	• Courage • Mercy	• Faculty • Department chairs • Students • Staff
**Student observes intern writing SOAP notes on patients the intern has not seen in order to prepare for attending rounds**	• Honesty • Social responsibility • Professional responsibility • Competence • Compassion	• Courage • Mercy	• Faculty • Department chairs • Program directors • Residents • Students
**Student complains about a faculty member’s disruptive behavior: berating and residents, foul language, physically pushing residents out of the way. Faculty member is very kind to students.**	• Respect for others • Professional responsibility • Competence • Compassion	• Courage • Humility • Mercy	• Students • Faculty • Residents
**Student A asks Student B to cover for him by pretending he just stepped out when he leaves the hospital instead of staying overnight for call.**	• Honesty • Compassion • Professional responsibility • Social Responsibility	• Courage • Humility • Mercy	• Students • Residents

For each of the cases, the audience was asked to identify the professional attributes in play for each of the stakeholders, as well as the desired outcome of the behaviors (positively reinforced, negatively reinforced, minor sanction, major sanction), and how they should be addressed.

During 2010, we presented the work of the task force and demonstrated case scenarios and problem solving at college faculty, chair, and associate/assistant dean meetings, as well as to representative groups of students. The students felt strongly that they could appropriately evaluate faculty on professionalism if a ‘student-on-faculty’ evaluation was developed. In January 2011, the task force was asking for input from various college leadership committees about developing a student evaluation of faculty professionalism as well. After initial resistance, mostly by a few chairs, the faculty voted in an open meeting to support the task force development of a “student-on-faculty” professionalism evaluation during clinical clerkships since ‘feedback’ on performance rated highly in the original faculty survey.

In May 2011, the task force leadership began to introduce the new virtues to a broader group of faculty and residents, including those at community campuses involving volunteer affiliate clinical faculty. In May of 2012, new virtues were introduced to 200 second-year medical students who would shortly be entering the intense clinical portion of their education. This was repeated in April of 2013 with greater details of the opportunity for students to evaluate the professionalism of any clinician educator for which they were assigned. Also, in April 2013, the task force leadership made presentations to affiliated residency programs regarding the implementation of the evaluation of residency faculty and residents by students. The evaluation form can be viewed at the following website: http://www.chmfacultyaffairs.msu.edu/professionalism/VPE.pdf.

### Dean’s Announcement

In May 2013, Dean Marsha Rappley sent an announcement to all faculty (including community volunteer faculty and residents) introducing “The Virtuous Professional: A System of Professional Development for Students, Residents and Faculty.” The announcement summarized the work of the task force on professionalism which included the three new virtues, the six professional responsibilities, and the process of professional growth. It also informed them that professionalism would soon be part of the evaluation of not only all learners, but also all clinical teachers in our entire community-based system.

## Conclusions and Next Steps

Now in 2018, 10 years after reframing professionalism in the college, faculty and students continue to demonstrate remarkable acceptance of this model of virtues, responsibilities, and the system of professional development. This is evidenced by demonstration of thoughtful faculty and student participation in the written evaluation and feedback process, consistently positive evaluations of presentations on the “Virtuous Professional” system by faculty and students, and ongoing thoughtful discourse and embracement of its stated values and goals.

As a community-based medical school with students spread across seven different areas of the state and with volunteer faculty continually entering and leaving the system, we needed a way to insure that our students and the faculty understood the meaning of professionalism in the same way, and aspired toward the same virtues. While some advocate for a medical professionalism curriculum in which faculty demonstrate and role model the virtues for students and residents, we report the actual reframing and adoption of a comprehensive approach where all members of the learning community are viewed as professionals who aspire to be virtuous ( MacIntyre 1984, Brody and Doukas 2014, Karches and Sulmasy 2016). Our approach could serve as a model for other institutions who seek to align faculty and student professionalism ( Egener, McDonald et al. 2012).

Along with dialogue and reflection, constructive feedback that travels both directions between students and faculty members is the logical next step to our model. Recent literature suggests that collecting and delivering this feedback is one of the greatest challenges facing medical educators ( Lucey, Levinson et al. 2016). The task force was able to devise a method for students to evaluate faculty and resident professionalism in the same manner by which faculty and residents evaluate students. This evaluation of faculty professionalism will provide an opportunity to freshly address the “hidden curriculum.” Recalling one of the fundamental reasons for reframing our “Virtuous Professional” system, the evaluations of faculty will provide a mechanism for students to call out both outstanding professionalism in faculty members, as well as lapses. A future manuscript will describe that process, and the somewhat surprising results we observed with the faculty professionalism evaluation. While others have focused on clinical events ( Hickson 2007), our evaluation focuses on student and faculty interactions.

Future opportunities to build upon the work we have done so far include a faculty development program focused on addressing professionalism issues in colleagues and providing concrete data about faculty professionalism for use at the time of annual performance review.

## Take Home Messages

• Form a broad-based task force of faculty with student and resident physician involvement.
• Survey all faculty on their knowledge and level of engagement in professionalism education for the college.
• Disseminate survey results in multiple venues engaging discussion of faculty, students, resident physicians, and administrative leaders.
• Reframe the curriculum and standards based on feedback to be all inclusive, relevant, and aspirational for the entire college and learning community based on institution selected virtues (Courage, Humility, and Mercy), not just rules of performance.
• Provide mutual professional performance feedback to faculty and students.

## Notes On Contributors

All authors were members of the 2008 Task Force to Reframe Professionalism in the College of Human Medicine

William C. Wadland, M.D., M.S. is Professor Emeritus, former Senior Associate Dean for Faculty Affairs and Development, and former Chairperson of Family Medicine at Michigan State University College of Human Medicine, East Lansing, Michigan. He chaired the Task Force to Reframe Professionalism.

Margaret E. Thompson, M.D. is Associate Dean for Academic Affairs and an Associate Professor of Family Medicine at Michigan State University College of Human Medicine, Grand Rapids, Michigan.

Donna D. Mulder is Director of Faculty Affairs and Development at Michigan State University College of Human Medicine, East Lansing, Michigan.

Tom Tomlinson, Ph.D. is Professor and Director of the Center for Ethics and Humanities at Michigan State University College of Human Medicine, East Lansing, Michigan.

Steven E. Roskos, M.D. is Associate Professor of Family Medicine at Michigan State University College of Human Medicine, East Lansing, Michigan.

John P. Foglio, D.Min. is Emeritus Assistant Professor of Family Medicine at Michigan State University College of Human Medicine, East Lansing, Michigan.

John B. Molidor, Ph.D. is Professor of Psychiatry and the Office of Medical Education Research and Development at Michigan State University College of Human Medicine. He also serves as the Assistant Dean and Chief Executive Officer of Michigan State University Flint Area Medical Education, Flint, Michigan.

Janet R. Osuch, M.D., M.S. is Professor of Surgery and Epidemiology and Assistant Dean of Preclinical Curriculum at Michigan State University College of Human Medicine, East Lansing, Michigan.
