# Supplementary material for: Reframing Professionalism: The Virtuous Professional
Source: MedEdPublish (2016). 2018 Apr 3;7:74. [Version 1] doi: 10.15694/mep.2018.0000074.1 (PMC10711981; doi:10.15694/mep.2018.0000074.1)
Supplement: Peer review history

# Reviewer report: Reviewer response for version 1

**DOI:** 10.21956/mep.19539.r28938

**Published:** 2018-05-22

**Authors:** Sateesh Babu Arja

**Affiliations:** 1 Avalon University School of Medicine

This review has been migrated. The reviewer awarded 5 stars out of 5 I thoroughly enjoyed reading this paper and this paper definitely adds to the existing literature on professionalism framework. This paper can be very useful in guiding the small medical schools or even the medical schools which are established recently to set their goals and objectives in the professionalism competency area which is one of the key attribute required for the future physicians. Professionalism is one of the key competencies required by physicians as published in the competency framework by either ACGME or CanMEDs.I also enjoyed reading the historical development of professionalism framework at the college of human medicine. I liked the idea of trickling down the professionalism from faculty members to students. Here clinicians and faculty members act as role models for students. This paper is useful for all healthcare professionals, faculty members, and academic administrators. Even the regulatory/accreditation bodies are stressing the importance of general professional education in the medical programs.

Reviewer Expertise:

NA

No decision status is available

# Reviewer report: Reviewer response for version 1

**DOI:** 10.21956/mep.19539.r28940

**Published:** 2018-04-08

**Authors:** Subha Ramani

**Affiliations:** 1 Harvard Medical School, Brigham and Women's Hospital

This review has been migrated. The reviewer awarded 5 stars out of 5 I enjoyed reading the paper very much and appreciate the authors for this very innovative approach to tackle the hidden curriculum. Professionalism initiatives at educational institutions are mostly directed towards learners and not faculty. While it is generally acknowledged that learners pick up unprofessional behaviors from negative role-model faculty, few institutions directly tackle these faculty behaviors or implement curricula to address this topic. This program is especially innovative as students' input was incorporated in program design. The paper is well written, well referenced and the curriculum can be applied at other institutions worldwide. It is also most relevant when programs such as these are mapped to the institutional mission and vision.All health professions educators will benefit from reviewing this paper.

Reviewer Expertise:

NA

No decision status is available

# Reviewer report: Reviewer response for version 1

**DOI:** 10.21956/mep.19539.r28939

**Published:** 2018-04-07

**Authors:** Gary D. Rogers

**Affiliations:** 1 Griffith University

This review has been migrated. The reviewer awarded 4 stars out of 5 This is an interesting and readable paper that provides an historical perspective on the development of a framework and processes for the promotion of professionalism among both students and staff. Although it is focused only on a single institution and the solutions that were found could not be transplanted without modification to other settings, it nontheless makes interesting reading for all educators interested in this complex area of health professional education practice.

Reviewer Expertise:

NA

No decision status is available

# Reviewer report: Reviewer response for version 1

**DOI:** 10.21956/mep.19539.r28937

**Published:** 2018-04-06

**Authors:** Trevor Gibbs

**Affiliations:** 1 AMEE

This review has been migrated. The reviewer awarded 4 stars out of 5 A very interesting and informative paper, covering a very important issue. With the expansion of the curriculum, the growth of new medical schools and the wider dissemination of learning opportunities for students, the translation of what is written on paper to practice becomes even more difficult and monitoring of these activities greater. Professionalism sits firmly in this area.The school has embarked on a very interesting piece of research which I feel all involved in professionalism education need to read. I personally look forward to reading how this piece of research evolves

Reviewer Expertise:

NA

No decision status is available
